# Astragaloside IV attenuates hypoxia-reoxygenation-induced endothelial senescence and vascular inflammation by modulating the NOTCH1/VCAM-1 axis

**DOI:** 10.3389/fnagi.2026.1855522

**Published:** 2026-08-28

**Authors:** Dehui Huang, Rongjing Zhou, Jun Ling

**Affiliations:** Ningbo Municipal Hospital of Traditional Chinese Medicine (TCM), Affiliated Hospital of Zhejiang Chinese Medical University, Ningbo, China

**Keywords:** Alzheimer's disease, Astragaloside IV, endothelial senescence, NOTCH1, senescence-associated secretory phenotype, VCAM-1

## Abstract

**Objective:**

Alzheimer's disease (AD) progression involves cerebral microvascular endothelial cell senescence induced by brain hypoperfusion, which contributes to blood-brain barrier (BBB) dysfunction. While Notch1 signaling is known to exacerbate endothelial senescence and neuroinflammation via vascular cell adhesion molecule-1 (VCAM-1), the mechanism by which it mediates hypoxia-induced endothelial aging in AD remains unclear. Furthermore, although Astragaloside IV (AS-IV) has been shown to alleviate cerebral hypoperfusion in AD, whether it acts by directly modulating the Notch1/VCAM-1 axis is unknown. This study aimed to determine whether AS-IV mitigates endothelial senescence and AD pathology by modulating the Notch1/VCAM-1 pathway.

**Methods:**

Transcriptomic analysis of AD patient data and network pharmacology identified *NOTCH1* as a key target. Molecular docking and 100-ns molecular dynamics simulations (using Desmond) characterized AS-IV–Notch1 interactions. Human brain microvascular endothelial cells (HBMECs) were subjected to hypoxia-reoxygenation (HR) to model AD-associated hypoperfusion. Cells were treated with AS-IV (25, 50, or 100 μM) or N-acetylcysteine (NAC) as a positive antioxidant control. Notch1 signaling was experimentally modulated using a recombinant decoy receptor to simulate signaling blockade, creating a contrast with HR-induced overactivation. Assessments included CCK-8 assays, MDA and ROS detection, qPCR, and Western blotting.

**Results:**

*NOTCH1* expression was upregulated in AD patients. Molecular docking and dynamics simulations predicted a stable binding mode between AS-IV and Notch1. HR exposure significantly increased oxidative stress and upregulated senescence markers (p16, p21, and p53), proinflammatory senescence-associated secretory phenotype (SASP) factors (IL-6, IL-1β, and TNF-α), and Notch1/VCAM-1 expression. Conversely, AS-IV treatment improved cell viability, reduced oxidative stress (with efficacy comparable to NAC), and downregulated Notch1/VCAM-1, senescence, and SASP markers. Notably, perturbing Notch signaling, either through HR-induced overactivation or decoy receptor-mediated blockade, exacerbated the senescent phenotype. Importantly, AS-IV co-treatment effectively rescued cellular damage induced by both forms of Notch pathway dysregulation.

**Conclusion:**

AS-IV alleviates HR-induced endothelial senescence by modulating the Notch1/VCAM-1 axis. Our data suggest that AS-IV does not merely inhibit Notch1 but acts by modulating its signaling toward a protective equilibrium, thereby attenuating endothelial senescence and inflammation associated with the restoration of Notch1/VCAM-1 signaling balance. These findings highlight the potential of AS-IV as a therapeutic candidate for AD vascular pathology.

## 1 Introduction

Alzheimer's disease (AD) is a progressive neurodegenerative disorder characterized by insidious onset and complex pathogenic mechanisms, posing significant challenges for treatment (Lane et al., 2018; Long and Holtzman, 2019). As a pressing global public health concern exacerbated by an aging population (Nichols et al., 2022; Livingston et al., 2020), AD remains difficult to treat effectively due to its multifactorial etiology and the challenges of early diagnosis (Scheltens et al., 2021). Beyond classical amyloid-β(Aβ) and tau pathologies, accumulating evidence highlights the critical role of cerebral microvascular dysfunction in AD progression. This dysfunction, manifesting as blood-brain barrier (BBB) disruption and endothelial senescence, is not merely a secondary phenomenon but an early driver of neuroinflammation, synaptic impairment, and cognitive decline (Montagne et al., 2020). For instance, endothelial injury can promote cerebral microvascular dysfunction and early BBB disruption, partly through mechanisms involving tau hyperphosphorylation (Karakaya et al., 2024; Wu et al., 2024). Despite these advances, the precise molecular pathways linking endothelial senescence to AD pathology, as well as corresponding therapeutic strategies, remain incompletely understood.

Endothelial senescence is not merely a state of proliferative arrest but is also characterized by a proinflammatory secretome known as the senescence-associated secretory phenotype (SASP) (Giuliani et al., 2023; Wang et al., 2024). The release of SASP factors, such as interleukin-1β (IL-1β), interleukin-6 (IL-6), and tumor necrosis factor-α (TNF-α), creates a deleterious microenvironment that perpetuates vascular dysfunction and exacerbates neuronal damage (Di Micco et al., 2021; Bussian et al., 2018). Critically, the production of SASP is governed by specific upstream signaling pathways. Among these, the Notch1 signaling pathway has been directly implicated in the induction and maintenance of SASP in endothelial cells under stress conditions (Liu et al., 2024; Park et al., 2021). Notch1 activation is upregulated during cerebral hypoperfusion and ischemia, where it impairs vascular remodeling and compromises BBB integrity (Li et al., 2009). In endothelial cells, heightened Notch1 signaling promotes a proinflammatory and senescent niche within the neurovascular unit, largely through the transcriptional induction of vascular cell adhesion molecule-1 (VCAM-1) (Liu et al., 2024; Park et al., 2021). Thus, Notch1 serves as a pivotal upstream regulator linking hypoperfusion to chronic vascular inflammation and senescence, making it a promising therapeutic target.

Astragaloside IV (AS-IV), a bioactive triterpenoid saponin derived from *Astragalus membranaceus*, is renowned for its antioxidant, anti-inflammatory, and neuroprotective properties (Liang et al., 2023). Notably, AS-IV exhibits sufficient blood-brain barrier permeability and has been shown to alleviate cognitive impairment and endothelial injury in various AD models (He et al., 2023). Previous studies demonstrated that AS-IV mitigates cognitive decline by attenuating endothelial injury and regulating neuroinflammation during AD progression (Wang et al., 2020; Li et al., 2018). Given the established vascular protective effects of AS-IV and the central role of the Notch1/VCAM-1 axis in driving vascular senescence, we hypothesized that AS-IV exerts its protective effects against AD-related vascular pathology, at least in part, by modulating this specific signaling axis.

To test this hypothesis, we employed a multi-level investigative approach. First, we integrated transcriptomic analysis of AD patient data with network pharmacology to identify *NOTCH1* as a key therapeutic target. Second, molecular docking and 100-ns molecular dynamics simulations were performed to predict and characterize the potential interaction between AS-IV and the Notch1 protein. Finally, these computational insights were functionally validated using a cellular model of hypoxia-reoxygenation (HR)-induced endothelial senescence. Since cerebral hypoperfusion is an early hallmark of AD that often precedes and occurs independently of Aβ deposition, the HR model serves as a well-established *in vitro* system to simulate these ischemic-hypoxic insults and the resulting oxidative stress. Instead of replicating Aβ-induced neuronal toxicity, this approach enables us to focus directly on how cerebrovascular endothelial senescence independently contributes to AD progression. We further utilized a recombinant Notch1 decoy receptor to perturb basal Notch signaling and assess whether AS-IV could attenuate the resulting endothelial phenotype. Our study provides a theoretical foundation for novel therapies targeting endothelial senescence in AD.

## 2 Materials and methods

### 2.1 Materials

Astragaloside IV (AS-IV; Cat. No. HY-N0431, purity ≥99.93%) and N-acetyl-L-cysteine (NAC; Cat. No. HY-B0215, purity ≥99.81%) were purchased from MedChemExpress (MCE, Monmouth Junction, NJ, United States). AS-IV was dissolved in dimethyl sulfoxide (DMSO) to prepare a 100 mM stock solution and stored at −20 °C. Primary antibodies against Notch1 (Cat. No. 3447T) and VCAM-1 (Cat. No. 14694S) were obtained from Cell Signaling Technology (Danvers, MA, United States). Recombinant human Notch1 Fc chimera protein (Cat. No. HY-P73742), consisting of the soluble extracellular domain fused to an Fc tag, was also purchased from MCE. This recombinant protein functions as a decoy receptor by sequestering endogenous Notch ligands, thereby inhibiting membrane-bound Notch1 activation. Cell culture consumables were sourced from Corning (Corning, NY, United States).

### 2.2 Transcriptomics analysis

Transcriptomic data from patients with Alzheimer's disease (AD) were retrieved from the Gene Expression Omnibus (GEO) database (Accession No. GSE122063). Differentially expressed genes (DEGs) were identified using the **limma** package in R software, and results were visualized via volcano plots and heatmaps. Functional enrichment analyses, including Gene Ontology (GO) and Kyoto Encyclopedia of Genes and Genomes (KEGG) pathways, were performed on the key DEGs using the bioinformatics platform (http://www.bioinformatics.com.cn) to elucidate relevant biological processes and molecular mechanisms.

### 2.3 Network pharmacology analysis

Potential targets of AS-IV were screened from the DrugBank (go.drugbank.com) and Traditional Chinese Medicine Systems Pharmacology (TCMSP) databases. Compounds were selected based on oral bioavailability (OB) ≥30% and drug-likeness (DL) ≥0.18. AD-related targets were collected from the GeneCards database (http://www.genecards.org) with a relevance score threshold of ≥10. Intersectional targets between AS-IV and AD were identified using Venn diagrams generated in Cytoscape 3.10.2. A protein-protein interaction (PPI) network was constructed using the STRING database (string-db.org) to identify core therapeutic targets.

### 2.4 Molecular docking analysis

The three-dimensional structure of human NOTCH1 (UniProt: P46531) was retrieved from the AlphaFold Protein Structure Database (AF-P46531-F1). PDB 3L95, an experimentally determined human Notch1 NRR structure, was used only as a structural reference and not as the docking receptor. The 3D structure of the AS-IV ligand was obtained from the ChEMBL database. Molecular docking was performed using AutoDock Vina with an exhaustiveness value of 8. A pocket near Arg1937 and Tyr1938 in the full-length NOTCH1 sequence was selected from the energetically highest-ranked cluster obtained by blind global docking. Because these positions map outside the extracellular NRR in the canonical sequence, no NRR- or S2-site-specific mechanistic inference was made from this pose. The grid box was defined with center coordinates (*x* = 12.5, *y* = 18.3, *z* = 22.1) Å and dimensions of 40 × 40 × 40 Å. Post-docking analysis and structural visualization were performed using PyMOL (v2.5) to evaluate binding affinity and interaction patterns of the AS-IV–Notch1 complex.

### 2.5 Molecular dynamics simulation

All-atom molecular dynamics simulations were performed using the Desmond module (Schrödinger, LLC, New York, NY, United States) to validate the optimal AS-IV–Notch1 docking pose. The OPLS4 force field was applied for protein and ligand parameterization, and the system was solvated in a cubic SPC/E water box with 10 Å padding. Physiological ionic conditions were maintained by adding 0.15 M NaCl, and system charge was fully neutralized. Energy minimization was conducted for 50,000 steepest-descent steps to eliminate steric clashes. The system was equilibrated through 50,000-step NVT and NPT ensemble simulations sequentially, with positional restraints applied to all heavy atoms. Unrestrained production simulation was run for 100 ns at 300 K and 1 bar with a 2 fs timestep. The Prime MM-GBSA method with the VSGB 2.0 solvation model and OPLS4 force field was used to calculate binding free energy. A total of 1,000 trajectory frames extracted from the stable 50–100 ns simulation period were used for averaging, yielding an average ΔG_bind of −44.5 kcal/mol. Considering the hydrophobic triterpenoid scaffold of AS-IV, persistent hydrogen bonding interactions, and the inherent estimation bias of the end-point MM-GBSA method for hydrophobic ligands, this high favorable binding energy represents a computationally predicted thermodynamic preference rather than definitive experimental affinity. Trajectory analysis and interaction fingerprint characterization were performed using Maestro (v13.5).

### 2.6 Cell culture and treatment

Human brain microvascular endothelial cells (HBMECs) were purchased from Pricella Biotechnology (Wuhan, China) and cultured in DMEM supplemented with 10% fetal bovine serum (FBS) and 1% penicillin-streptomycin at 37 °C in a humidified atmosphere containing 5% CO_2_.

#### 2.6.1 Hypoxia-reoxygenation (HR) Model

To simulate cerebral hypoperfusion, an HR model was established. Cells were exposed to hypoxic conditions (94% N_2_, 1% O_2_, 5% CO_2_) in serum-reduced medium (1% FBS) for 12 h using a modular incubator chamber (Heal Force, Shanghai, China), followed by reoxygenation under normoxic conditions (37 °C, 5% CO_2_) in complete medium (10% FBS) for 8 h.

#### 2.6.2 Experimental Groups

HBMECs were randomly divided into six groups: (1) Control (normoxia); (2) Model (HR injury); (3–5) AS-IV treatment groups (25, 50, or 100 μM AS-IV administered during HR) (He et al., 2023; Wang et al., 2020; Zhu et al., 2019); (6) Positive control (100 μM NAC). NAC (100 μM) was selected as a positive antioxidant control based on its well-characterized ROS-scavenging activity and established use in endothelial oxidative stress models at this concentration (Marasinghe et al., 2025).

To investigate the role of Notch signaling, cells were pretreated with recombinant human Notch1 decoy receptor (R&D Systems, Cat. No. 1786-NR; 1 μg/mL), a concentration determined in our preliminary optimization experiments, for 1 h before HR induction, followed by treatment with or without concurrent AS-IV. For all groups, the final DMSO concentration was kept below 0.1% to exclude solvent effects.

### 2.7 Cell biochemical measurements

Following treatment, cells were harvested and lysed. Intracellular levels of malondialdehyde (MDA) and reactive oxygen species (ROS) were measured using commercial kits (Beyotime Institute of Biotechnology, Shanghai, China) according to the manufacturer's protocols. For ROS detection, cells were incubated with 10 μM DCFH-DA at 37 °C for 20 min in the dark. Fluorescence intensity was quantified using a microplate reader (BioTek Instruments, Winooski, VT, United States) at excitation/emission wavelengths of 488/525 nm.

Cell viability was determined using the CCK-8 assay. After corresponding treatments, 10 μL CCK-8 working solution was added to each well and incubated at 37 °C for 2 h. The absorbance at 450 nm was detected with a microplate reader to calculate relative cell viability.

### 2.8 Quantitative real-time PCR (qPCR)

Total RNA was extracted from HBMECs using TRIzol reagent, followed by reverse transcription to generate complementary DNA (cDNA). Quantitative real-time PCR was carried out with SYBR Green Master Mix on a real-time PCR instrument. Target genes included *NOTCH1, VCAM1, HPRT1, CDKN2A* (p16)*, CDKN1A* (p21)*, TP53* (p53), *IL1B, IL6*, and *TNF*. All forward and reverse primer sequences for these genes are listed in Supplementary Table S1. Amplification conditions included an initial denaturation at 95 °C for 10 min, followed by 40 cycles of 95 °C for 15 s and 60 °C for 1 min. Melting curve analysis was performed to verify primer specificity. Relative gene expression was calculated using the $2^{-\Delta \Delta {\text{C}}_{\text{t}}}$ method with *HPRT1* as the internal reference gene.

### 2.9 Western blot assay

Protein concentrations were determined using a BCA assay kit (Beyotime). Equal amounts of protein (30 μg/lane) were loaded for all samples. Target proteins (Notch1, VCAM-1) and the β-actin loading control were separated on parallel 10% SDS-PAGE gels and transferred to individual PVDF membranes, with identical electrophoresis and transfer conditions applied across gels. Membranes were blocked with 5% non-fat milk and incubated overnight at 4 °C with primary antibodies against Notch1 (1:400), VCAM-1 (1:500), or β-actin (1:2,000), respectively. After washing, membranes were incubated with HRP-conjugated secondary antibodies (1:2,000) for 2 h at room temperature. Protein bands were visualized using an enhanced chemiluminescence (ECL) system (Thermo Fisher Scientific, Waltham, MA, United States) and quantified using ImageJ software (NIH, Bethesda, MD, United States). Target protein levels were normalized to β-actin. Full-length uncropped Western blot membranes are provided in Supplementary Figures S1–S4 for data traceability.

### 2.10 Statistical analysis

**Figure 1:**
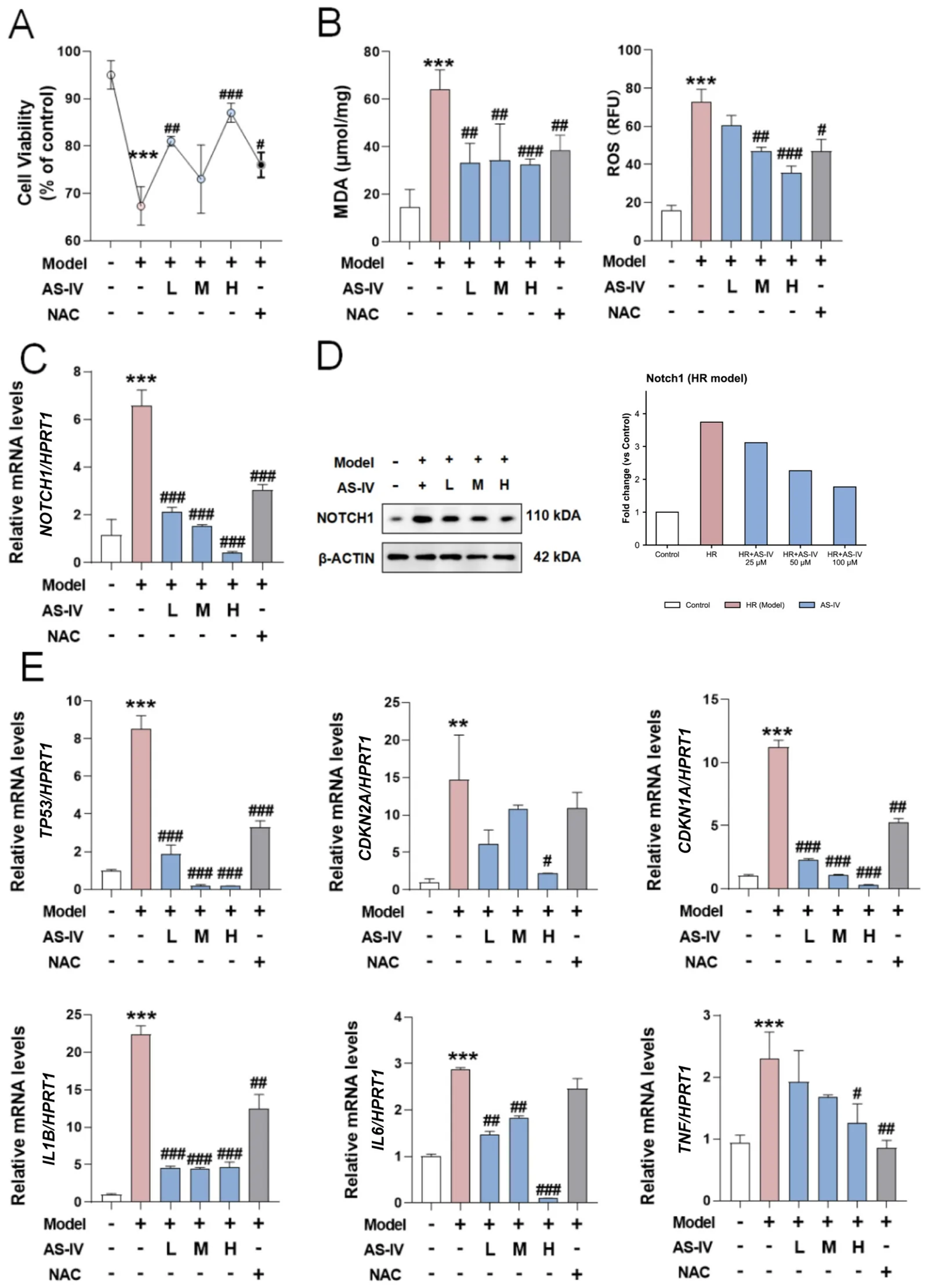
AS-IV attenuated oxidative stress and senescence-associated changes in HBMECs under HR, effects associated with reduced Notch1 expression. **(A)** The cell viability of HBMECs treated by HR stimulation, AS-IV in different doses (25, 50 and 100 μM) and NAC (100 μM). **(B)** The levels of MDA and ROS in HBMECs stimulated by HR, AS-IV and NAC (100 μM). **(C)** Relative expression of mRNA levels of *Notch1* was measured by qPCR and further normalized with *Hprt1*. **(D)** Western blot bands and quantitative bar graph for Notch1 protein, normalized to β-Actin. Blot images and quantification were derived from a single independent experiment without additional biological replicates. **(E)** Relative expression of mRNA levels of *TP53, CDKN2A, CDKN1A, IL1B, IL6* and *TNF* were measured by qPCR and normalized to *HPRT1*. Statistical significance: **P < 0.01, ***P < 0.001, compared with the control group; ^#^*P* < 0.05, ^##^*P* < 0.01, ^###^*P* < 0.001, compared with the model group. Data were obtained from three independent biological replicates (*n* = 3); Western blot results are representative of one independent experimental trial (n=1, no biological replicates).

**Figure 2:**
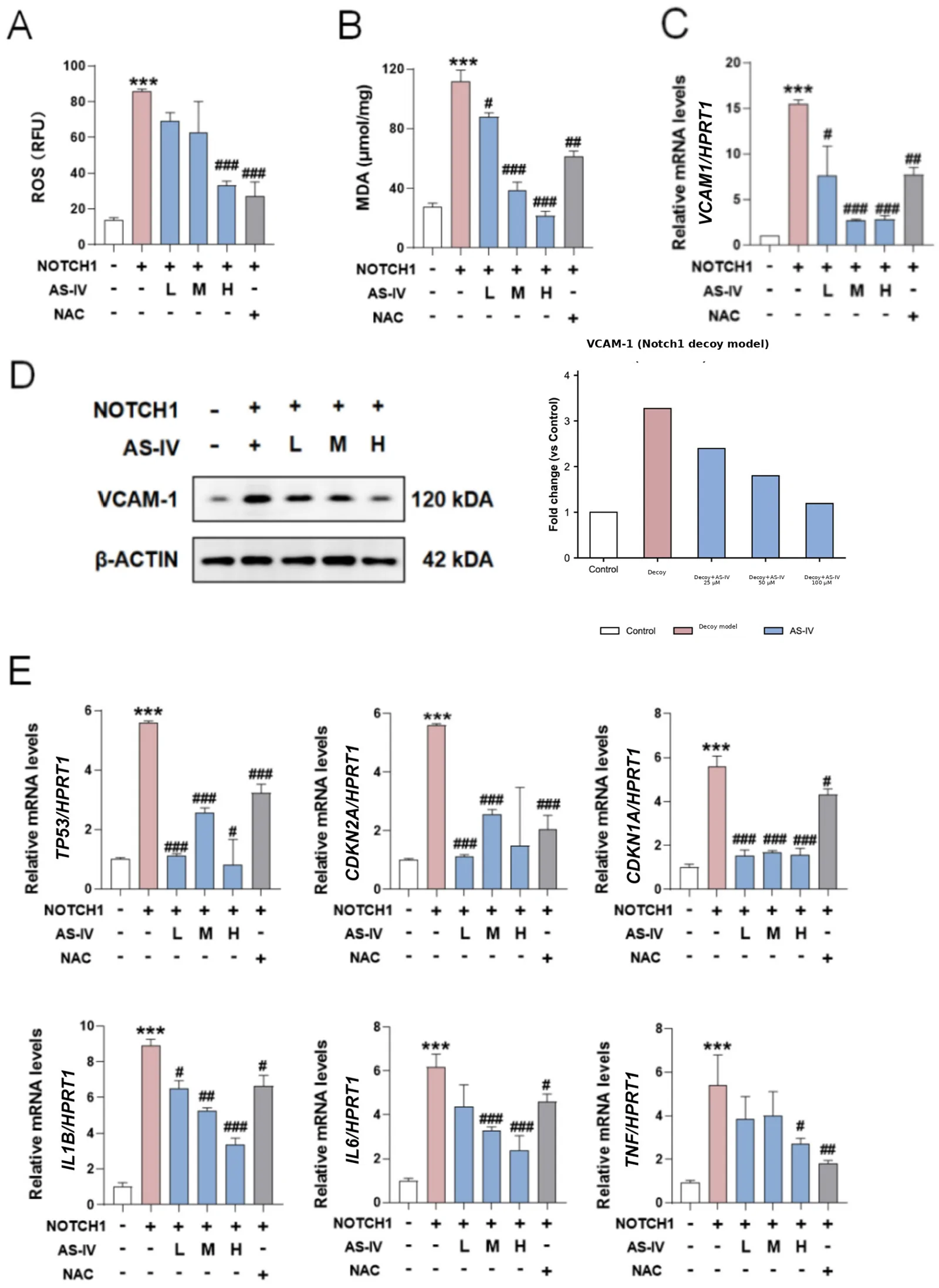
AS-IV alleviated HBMEC senescence induced by experimental Notch pathway inhibition via recombinant Notch1 decoy protein. **(A)** ROS and **(B)** MDA in HBMECs subjected to Notch1 pathway inhibition using recombinant Notch1 decoy protein, AS-IV and NAC (100 μM). **(C)** Relative expression of mRNA levels of *Vcam1* was measured by qPCR and further normalized with *Hprt1*. **(D)** Western blot bands and corresponding quantitative bar chart for VCAM-1 protein normalized to β-Actin; Western blot data were obtained from a single experimental run without biological replicates. **(E)** Relative mRNA levels of *TP53, CDKN2A, CDKN1A, IL1B, IL6* and *TNF* were measured by qPCR and normalized to *HPRT1*. All three AS-IV concentrations relieved the elevation of oxidative stress and senescence-related transcripts, with gradual VCAM-1 protein suppression observed as AS-IV concentration increased, while non-uniform inhibitory effects were seen for SASP mRNAs. Statistical significance: **P < 0.01, ***P < 0.001, compared with the control group; ^#^*P* < 0.05, ^##^*P* < 0.01, ^###^*P* < 0.001, compared with the group treated with recombinant Notch1 inhibitor. Data were obtained from three independent biological replicates (n=3); Western blot results are representative of one independent experimental trial (n=1, no biological replicates). The symbol “**” indicates *P* < 0.01 compared with the control group.

Unless otherwise specified, data are presented as mean ± standard deviation (SD) from at least three independent experiments. Western blot densitometry in Figures 1D, 2D represents one independent experimental run (*n* = 1) and was not subjected to inferential statistical analysis. Statistical comparisons among multiple groups were performed using one-way analysis of variance (ANOVA) followed by Tukey's *post-hoc* test using GraphPad Prism 8.0.2 (GraphPad Software, San Diego, CA, United States). A *P*-value < 0.05 was considered statistically significant. Exact *P*-values are indicated in the figure legends where applicable.

## 3 Results

### 3.1 Analysis of key differential genes in Alzheimer's Disease

**Figure 3:**
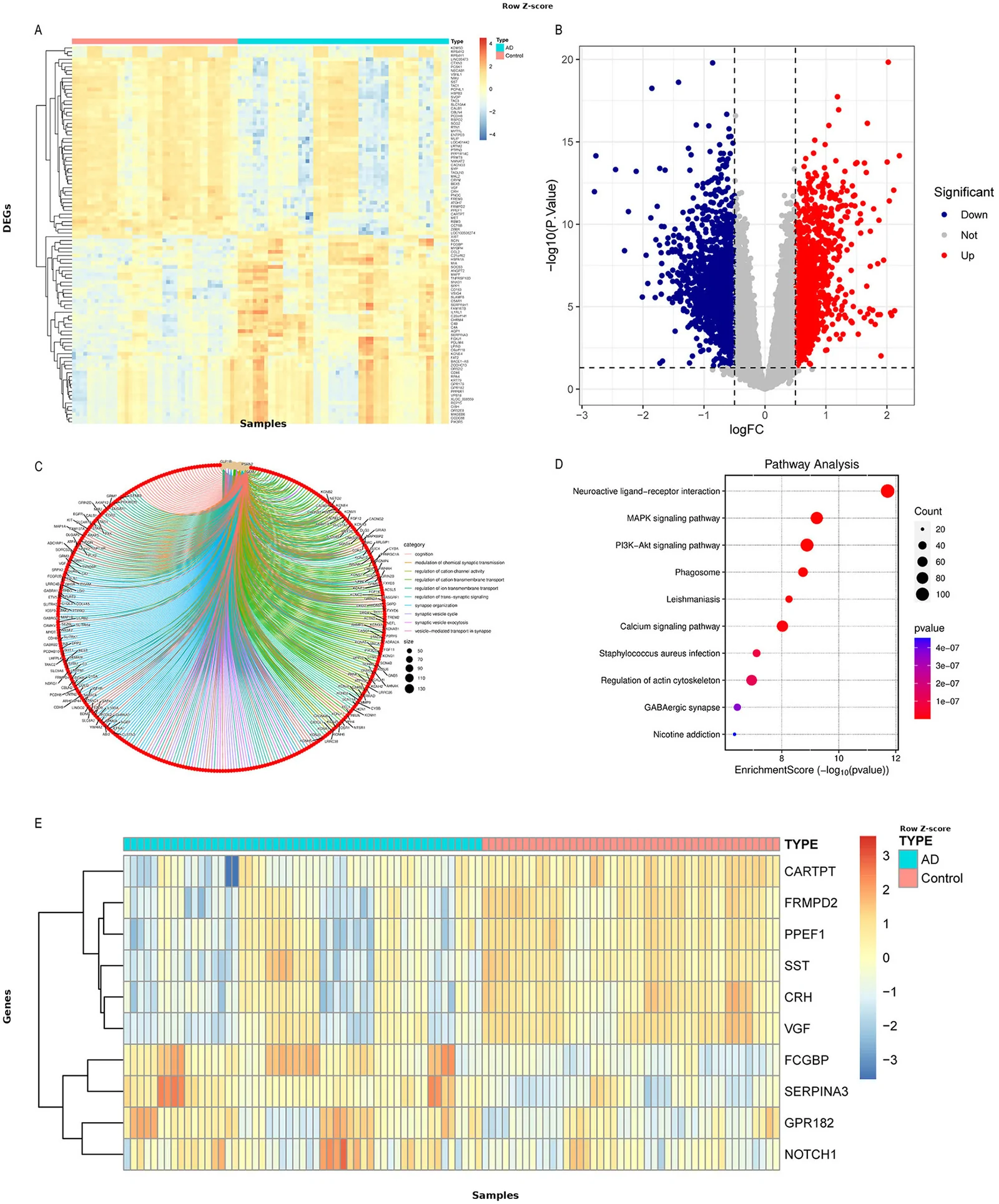
Transcriptomic analysis of differentially expressed genes (DEGs) from AD and control bulk brain tissues based on dataset GSE122063. **(A)** Heatmap showing expression patterns of screened DEGs. **(B)** Volcano plot visualizing significantly upregulated and downregulated genes between two groups. **(C, D)** GO and KEGG functional enrichment analysis of DEGs. **(E)** Top 10 most significantly dysregulated genes, where NOTCH1 was identified as a core gene related to cerebral vascular function for subsequent mechanistic research. This bulk tissue dataset reflects global transcriptional changes in whole brain tissue and cannot prove endothelial-specific NOTCH1 alteration; further functional verification in HBMECs was conducted to characterize cerebral endothelial-related mechanisms.

To identify dysregulated pathways in AD, we analyzed transcriptomic data from the GEO dataset GSE122063, which contains bulk brain tissue samples from AD patients and healthy controls. Differentially expressed genes (DEGs; *P* < 0.05) between AD patients and controls were identified and visualized via heatmaps and volcano plots (Figures 3A, B). Gene Ontology (GO) and KEGG pathway analyses revealed that these DEGs were significantly enriched in processes related to intracerebral cellular communication (e.g., GABAergic synapse, neuroactive ligand-receptor interaction), cell cycle regulation (e.g., calcium signaling, actin cytoskeleton regulation), and inflammatory pathways (e.g., MAPK and PI3K-AKT signaling) (Figures 3C, D). Among the top 10 most significantly altered genes, enrichment was observed in two functional clusters: neuropeptide activity (*CARTPT, FRMPD2, PPEF1, SST, CRH*, and *VGF*) and processes associated with cell proliferation and vascular homeostasis (*FCGBP, SERPINA3, GPR182*, and *NOTCH1*) (Figure 3E). Although this dataset reflects global brain changes, the identification of *NOTCH1*, a gene critical for both neurodevelopment and cerebrovascular function, among the top DEGs prompted us to investigate it as a potential mediator linking broader AD pathology to specific vascular dysfunction. Importantly, independent single-cell RNA-seq evidence from Mathys et al. (2019) provides valuable cell-type context but does not, on its own, establish endothelial-specific NOTCH1 upregulation in this study.

### 3.2 Network pharmacology and molecular docking predict AS-IV binding to Notch1

**Figure 4:**
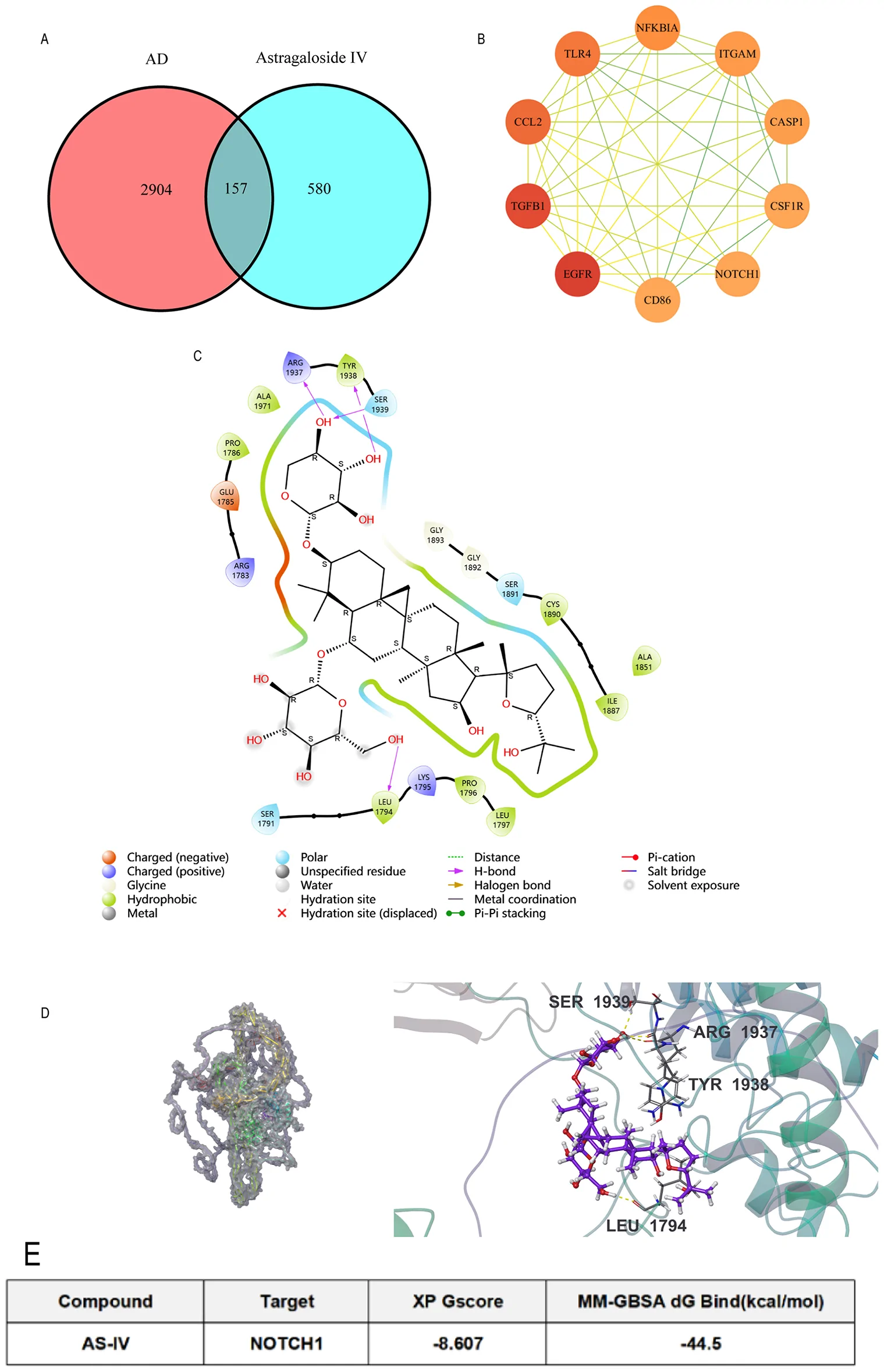
Pharmacological and molecular docking analysis of AS-IV on Notch1 during AD. **(A)** The venn diagram of targets of AS-IV and AD. **(B)** The protein-protein interaction network of top 10 genes in AS-IV and AD. **(C)** The 2D and **(D)** 3D visual ligand interaction diagrams and active binding sites of the predicted docking complexes of AS-IV and Notch1. **(E)** The molecular docking score of AS-IV and Notch1.

To explore the therapeutic mechanism of AS-IV in AD, we performed a network pharmacology analysis integrating AS-IV targets (from TCMSP and DrugBank) and AD-related targets (from GeneCards). Venn diagram analysis identified 157 overlapping targets between AS-IV and AD (Figure 4A). Subsequent protein-protein interaction (PPI) network analysis refined these intersections to 10 core hub genes (Figure 4B). Notably, *NOTCH1* emerged as a central hub connecting the pharmacological profile of AS-IV to AD pathology. Given its pivotal position and established role in AD-associated vascular dysfunction, we evaluated the direct interaction between AS-IV and Notch1 using molecular docking. The simulations predicted that AS-IV binds stably within a surface pocket of the Notch1 protein, engaging key residues (e.g., Arg1937, Tyr1938) located near regions critical for receptor activation and downstream signaling (Figures 4C, D). The complex exhibited a favorable docking score of −8.607 kcal/mol (Figure 4E). Detailed analysis indicated that this interaction is stabilized by hydrophobic contacts (e.g., with Pro1796, Leu1797) and multiple hydrogen bonds (e.g., with Arg1937, Tyr1938).

### 3.3 Molecular dynamics simulation suggests a stable binding mode

**Figure 5:**
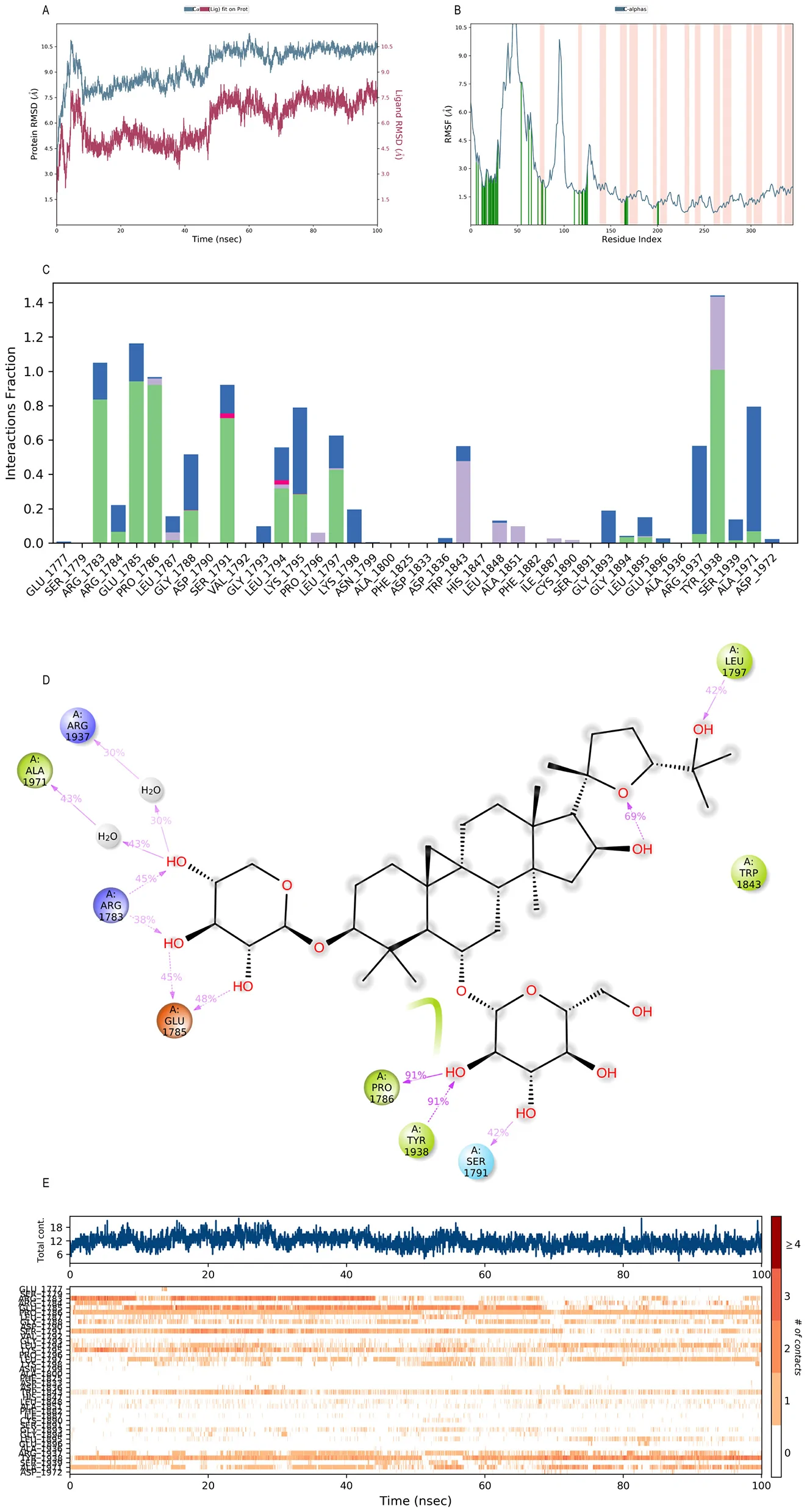
The molecular dynamics simulation between AS-IV and Notch1. **(A)** The RMSD and **(B)** RMSF plots of the AS-IV–Notch1 complex throughout the 100-ns MD simulation. **(C)** The interaction fraction and **(D)** binding sites of AS-IV-Notch1 complex. **(E)** Timeline of specific interactions (hydrogen bonds, hydrophobic contacts) between AS-IV and Notch1 throughout the 100-ns simulation.

**Figure 6:**
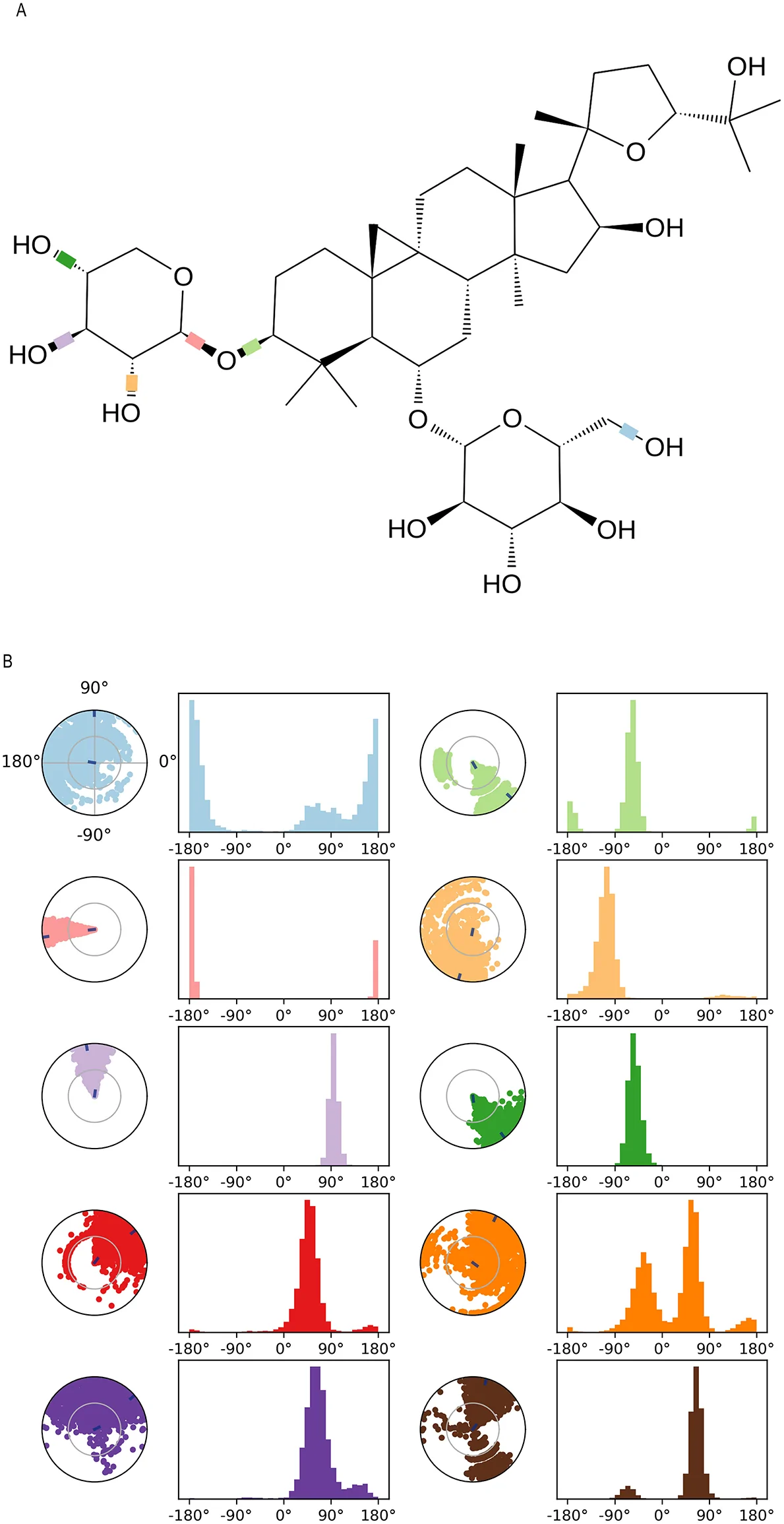
Torsional analysis of AS-IV during the MD simulation. **(A)** AS-IV structure with rotatable bonds highlighted. **(B)** Torsional-angle distributions of the selected rotatable bonds during the AS-IV–Notch1 MD simulation.

To validate the stability of the predicted AS-IV–Notch1 interaction, we conducted 100-ns all-atom molecular dynamics (MD) simulations. The root-mean-square deviation (RMSD) of the protein-ligand complex plateaued after approximately 50 ns, indicating system equilibration (Figure 5A). Root-mean-square fluctuation (RMSF) analysis revealed flexible regions, particularly within residue ranges 25–70 and 85–100, suggesting potential induced-fit adjustments upon ligand binding (Figure 5B). Interaction fraction analysis identified key residues (Arg1783, Glu1785, and Tyr1938, etc.) that maintained persistent hydrogen bonds and hydrophobic contacts throughout the simulation (Figures 5C, D). A timeline of specific contacts confirmed the sustained engagement of AS-IV with these residues over the 100-ns trajectory (Figure 5E). Furthermore, detailed molecular interaction diagrams illustrated the specific torsional conformations and residue engagements stabilizing the complex (Figure 6). The average binding free energy (Δ*G*_*bind*_), calculated via MM-GBSA over the stable trajectory, was −44.5 kcal/mol, indicating a computationally favorable interaction rather than confirming experimental binding affinity. These findings provide a physicochemical basis for the hypothesis that AS-IV forms a stable complex within the Notch1 binding pocket, thereby modulating its signaling activity.

### 3.4 AS-IV alleviates HR-induced endothelial senescence and oxidative stress

Given the critical role of cerebral microvascular endothelial dysfunction in AD and the involvement of Notch1 in senescence, we employed a human brain microvascular endothelial cell (HBMEC) model subjected to hypoxia-reoxygenation (HR) to mimic AD-related ischemic stress. First, we assessed cell viability and oxidative damage. AS-IV treatment (25, 50, and 100 μM) significantly improved cell viability compared to the HR model group. Notably, concentrations of 25 μM and 100 μM exhibited robust protective effects (*P* < 0.01), whereas 50 μM showed a more modest but still significant benefit (*P* < 0.05), suggesting a potentially non-linear dose-response relationship (Figure 1A). Furthermore, AS-IV significantly reduced HR-elevated levels of malondialdehyde (MDA) and reactive oxygen species (ROS), with antioxidant efficacy comparable to the positive control, N-acetylcysteine (NAC) (Figure 1B). To link this protection to our predicted target, we examined *NOTCH1* expression. qPCR analysis confirmed that HR significantly upregulated *NOTCH1* mRNA, an effect markedly suppressed by AS-IV treatment (Figure 1C). Western blot analysis from a single independent run showed a consistent trend of elevated Notch1 protein levels after HR exposure, with reduced levels observed following AS-IV treatment (Figures 1C, D). Consequently, AS-IV significantly attenuated HR-triggered elevation of cell cycle arrest and senescence-associated transcripts (*CDKN2A, CDKN1A, TP53*) and pro-inflammatory SASP-related genes (*IL1B, IL6, TNF*) at the transcriptional level (*P* < 0.001; Figure 1E). These results indicate that AS-IV alleviates HR-induced endothelial senescence-associated phenotype and inflammation, effects associated with suppression of Notch1 overexpression.

### 3.5 AS-IV alleviates endothelial senescence induced by notch signaling blockade

To further elucidate the functional role of Notch1 signaling, we perturbed its homeostasis by applying a recombinant Notch1 decoy receptor to block endogenous signaling in HBMECs. Surprisingly, disruption of basal Notch signaling also triggered a pronounced cellular stress response, evidenced by significantly elevated oxidative stress (MDA and ROS levels) (Figures 2A, B). Decoy receptor treatment led to a marked increase in *VCAM1* mRNA and senescence/SASP transcript levels (Figures 2C, E). A concordant trend of elevated VCAM-1 protein abundance was observed in a single Western blot experiment, with a dose-dependent reduction seen following AS-IV co-treatment (Figure 2D). These findings indicate that both excessive activation (induced by HR) and insufficient signaling (induced by decoy blockade) of the Notch1 pathway are detrimental, promoting a senescent and pro-inflammatory phenotype. Importantly, co-treatment with AS-IV effectively rescued the cellular damage induced by Notch signaling blockade. All three tested concentrations reduced oxidative stress, VCAM-1 and senescence-related markers, with gradual suppression of VCAM-1 protein observed as AS-IV concentration rose, while non-uniform suppressive effects were seen for SASP transcripts (Figures 2A–E). These rescue experiments demonstrate that AS-IV does not simply inhibit Notch1; rather, it attenuates the endothelial senescent and pro-inflammatory phenotype by modulating the Notch1/VCAM-1 axis, an effect associated with the restoration of signaling equilibrium.

## 4 Discussion

Cerebrovascular dysfunction, particularly BBB disruption and microvascular endothelial senescence, is increasingly recognized as a critical contributor to AD pathology (Yue et al., 2024; Katusic et al., 2024; Graves and Baker, 2020). This study investigated the protective effects of AS-IV against endothelial injury and senescence using an *in vitro* HR stress model. Consistent with its reported antioxidant and anti-inflammatory properties (He et al., 2023; Chen et al., 2021), our current results show that AS-IV alleviates HR-induced endothelial senescence via regulating the Notch1/VCAM-1 signaling axis.

The Notch1 signaling pathway plays a complex, context-dependent role in vascular biology (Fernandez-Chacon et al., 2021). While classically characterized as a mechanosensor, our data reveal that Notch1 is also markedly upregulated by metabolic insults such as HR. Furthermore, our observation of *NOTCH1* upregulation in bulk AD brain tissue should not be interpreted as endothelial-specific; the cited single-cell study provides cell-type context but does not directly establish NOTCH1 elevation in brain microvascular endothelial cells in AD (Mathys et al., 2019). We propose that this aberrant activation under ischemic/hypoxic stress serves as a critical link between vascular injury and the senescent endothelial phenotype. Mechanistically, sustained Notch1 signaling triggers the cleavage of the receptor, releasing the Notch intracellular domain (NICD), which translocates to the nucleus to directly promote *VCAM1* transcription (Verginelli et al., 2015). We did not test cleaved Notch1/NICD protein levels in this work, which restricts direct quantification of pathway activation intensity. Elevated VCAM-1 then fosters leukocyte adhesion and a pro-inflammatory microenvironment, amplifying ROS production and activating DNA damage responses. This cascade ultimately leads to the upregulation of cell-cycle arrest proteins, including p53 and p21, hallmarks of the SASP (Mai et al., 2023; Machoń et al., 2025). Thus, Notch1 emerges as a central molecular switch connecting hypoperfusion to the endothelial inflammation and senescence that characterize the dysfunctional neurovascular unit in AD (Graves and Baker, 2020; Ting et al., 2023).

Therapeutic modulation of the Notch pathway remains clinically challenging due to its pleiotropic functions (Bao et al., 2023). Our integrated computational approach, combining network pharmacology and MD simulations, predicted a potentially stable binding mode between AS-IV and the Notch1 protein. The 100-ns MD simulation suggests that AS-IV may engage key residues within a binding pocket of Notch1 (Decherchi and Cavalli, 2020), potentially altering the receptor conformation to prevent pathological hyperactivation. Experimentally, we observed that AS-IV not only downregulates HR-induced Notch1 protein expression but also functionally regulates Notch1 pathway activity, counteracting the effects of exogenous pathway perturbation. This dual, multi-layered regulation likely underlies its effective suppression of endothelial senescence. However, we note that our evidence is based on total Notch1 mRNA and protein levels; direct measurement of NICD and canonical downstream targets (HES1, HEY1) will be required to confirm pathway-level modulation.

A pivotal finding of this study is that AS-IV functions not as a unidirectional inhibitor, but as a homeostatic modulator of the Notch1/VCAM-1 axis. Our data reveal a Goldilocks effect in Notch1 signaling within the brain microvasculature: while HR-induced pathological hyperactivation triggers the VCAM-1-mediated SASP, experimental sequestration of Notch ligands via a decoy receptor similarly exacerbates endothelial senescence. This suggests that a basal level of Notch1 activity is indispensable for vascular survival (Fernandez-Chacon et al., 2021). Intriguingly, AS-IV demonstrated the capacity to rescue both extremes of signaling disturbance. Unlike synthetic gamma-secretase inhibitors that cause off-target toxicity by complete blockade, AS-IV appears to recalibrate Notch1 signaling toward a protective equilibrium (Andersson and Lendahl, 2014; Zhdanovskaya et al., 2021). This homeostatic modulation, if confirmed by genetic loss- and gain-of-function experiments, may represent a novel therapeutic paradigm for mitigating AD-related vascular aging, shifting the focus from simple pathway suppression to the restoration of cellular resilience.

Of note, the protection of cell viability by AS-IV did not follow a strict linear dose-response, with 25 μM and 100 μM showing more pronounced effects than 50 μM. Such non-linear patterns are not uncommon for natural compounds and may reflect a non-linear pharmacological relationship (Forman et al., 2014; Pande and Raisuddin, 2022). In contrast, VCAM-1 protein expression showed a gradual downward trend with increasing AS-IV concentrations, indicating divergent concentration sensitivity between inflammatory transcription and protein signaling regulation. Further studies with broader dose ranges and time-course analyses are warranted to fully elucidate this relationship.

Several limitations of this study warrant consideration. First, the acute hypoxia-reoxygenation model only simulates acute endothelial injury and cannot fully recapitulate the chronic and multifactorial cerebrovascular pathology of AD. Further validation using chronic cerebral hypoperfusion or AD transgenic animal models is needed. Second, although molecular docking and MD simulations predicted AS-IV–Notch1 binding, direct biophysical verification of their interaction is still required. Third, only total Notch1 protein was detected in this study; cleaved NICD levels were not measured, limiting the direct evaluation of Notch pathway activation. Fourth, this *in vitro* study lacks validation in human AD tissues, and further clinical sample verification is necessary to strengthen translational evidence. Fifth, endothelial senescence and SASP phenotypes were evaluated only by transcriptional markers. SA-β-gal staining and protein-level detection of SASP factors were not performed, restricting phenotypic and functional confirmation of senescence. Sixth, the current design cannot fully distinguish Notch1/VCAM-1-specific regulation from the general antioxidant effects of AS-IV. NAC-mediated protection suggests that non-specific ROS scavenging partially contributes to endothelial improvement, and future Notch-targeted intervention will help clarify pathway-specific mechanisms.

## 5 Conclusion

In conclusion, our study elucidates that AS-IV attenuates endothelial senescence by restoring the homeostasis of the *Notch1*/*VCAM-1* axis, supporting a working model associated with modulation of this signaling axis. AS-IV appears to function not as a simple inhibitor but as a homeostatic modulator that attenuates both pathological hyperactivation and detrimental suppression of Notch signaling, though genetic validation of this mechanism is needed. Given the growing recognition of vascular dysfunction as an active driver of AD pathology, therapeutic strategies that preserve neurovascular integrity are of paramount importance. AS-IV, with its potential to improve cerebrovascular function through this defined pathway, presents a promising candidate for AD therapy. Future research should include direct biophysical binding validation, genetic loss- and gain-of-function experiments, and *in vivo* AD model studies to establish the causal role of the Notch1/VCAM-1 axis in AS-IV's therapeutic effects.

## Data Availability

The original contributions presented in the study are included in the article/Supplementary material, further inquiries can be directed to the corresponding author.
