# Supplementary material for: Astragaloside IV attenuates hypoxia-reoxygenation-induced endothelial senescence and vascular inflammation by modulating the NOTCH1/VCAM-1 axis
Source: Front Aging Neurosci. 2026 Aug 28;18:1855522. doi: 10.3389/fnagi.2026.1855522 (PMC13561900; doi:10.3389/fnagi.2026.1855522)
Supplement: Supplementary file 1 [file Table_1.docx]

**Table S1. Primers used for quantitative real-time PCR.**

| **Gene** | **Forward Primer (5′→3′)** | **Reverse Primer (5′→3′)** | **Product Size**  **(bp)** |
| --- | --- | --- | --- |
| NOTCH1 | TGGGACCAACTGTGACATCA | GCACACTCGTTGATGTTGGT | 156 |
| VCAM1 | CTACAAGTCTACATCTCACCCA | TTCCAGAATCTTCCAGCCTC | 213 |
| TP53 | TGAGGTTCGTGTTTGTGCCTGC | CCATCAAGTGGTTTTTTCTTTTGC | 165 |
| CDKN2A | TGGGGAGTAGTATGGAGTTGGTGGT | CAACCCCAAACCACAACCATAA | 81 |
| CDKN1A | TTTGGCTCCCCTGTACCTTT | CCTTCCCCTTCCAGTCCATT | 192 |
| IL6 | TGCTCAGAGGTCAGACGCTTCAG | TCCTGGCTGCTGGTCACTGC | 86 |
| IL1B | TTCCCCAAGTGCTGCTTATT | AAGTTAAAACCGCTGTGGTCA | 149 |
| TNF | CTTCTCATTCCTGCTTGTGG | ATCTGAGTGTGAGGGTCTGG | 140 |
| HPRT1 | TGACACTGGCAAAACAATGCA | GGTCCTTTTCACCAGCAAGCT | 94 |
